# First Complete Genomic Sequence of a Rabies Virus from the Republic of Tajikistan Obtained Directly from a Flinders Technology Associates Card

**DOI:** 10.1128/genomeA.00515-17

**Published:** 2017-07-06

**Authors:** H. Goharriz, D. A. Marston, F. Sharifzoda, R. J. Ellis, D. L. Horton, T. Khakimov, A. Whatmore, K. Khamroev, A. N. Makhmadshoev, M. Bazarov, A. R. Fooks, A. C. Banyard

**Affiliations:** a Wildlife Zoonoses & Vector-Borne Diseases Research Group, Animal and Plant Health Agency, Surrey, United Kingdom; b Specialist Scientific Support Department, Animal and Plant Health Agency, Surrey, United Kingdom; c Bacterial Characterization Team, Animal and Plant Health Agency, Surrey, United Kingdom; d National Center for Veterinary Diagnostics, Dushanbe, Republic of Tajikistan; e School of Veterinary Medicine, University of Surrey, Guildford, United Kingdom; f Institute for Infection and Immunity, St. George's Hospital Medical School, University of London, London, United Kingdom

## Abstract

A brain homogenate derived from a rabid dog in the district of Tojikobod, Republic of Tajikistan, was applied to a Flinders Technology Associates (FTA) card. A full-genome sequence of rabies virus (RABV) was generated from the FTA card directly without extraction, demonstrating the utility of these cards for readily obtaining genetic data.

## GENOME ANNOUNCEMENT

Rabies is one of the most neglected zoonotic diseases, causing an estimated 60,000 human deaths annually (1). Accurate diagnostics, as with many viral diseases, relies on sample collection and reliable transportation, which can require maintenance of a cold chain to preserve the integrity of the sample for diagnostic evaluation. In rabies, areas endemic for the disease have been identified as a bottleneck to diagnosis. The use of Flinders Technology Associates (FTA) filter paper to preserve nucleic acids in samples has proven invaluable under field conditions for numerous pathogens, including lyssaviruses (2, 3). However, extraction of viral nucleic acid was performed before downstream applications, such as molecular diagnostics or phylogenetic analysis.

One hundred microliters of a clarified brain homogenate that tested positive at the National Centre for Veterinary Diagnostics (NCVD), Dushanbe, Tajikistan, by fluorescent antibody test (FAT) was placed onto an FTA card and allowed to air dry before transportation to the Animal and Plant Health Agency (APHA) for molecular characterization.

RNA was eluted from a 2-mm punch obtained from the FTA card in 50 µl of molecular-grade water on ice, with periodic agitation. The eluted nucleic acid was depleted of host genomic DNA (4), and double-stranded (ds) cDNA was synthesized using a random cDNA synthesis system (Roche). The ds cDNA was purified using AMPure XP magnetic beads (Beckman Coulter, Inc.) and 1 ng used for the Nextera XT DNA sample preparation kit (Illumina). A sequencing library was prepared and sequenced on an Illumina MiSeq with 2 × 150 bp paired-end reads according to standard Illumina protocols. The total reads (6,540,291) were mapped to a reference sequence (accession no. JQ944705) using BWA (version 0.7.5a-r405) (5) and was visualized in Tablet (6). A modified SAMtools/vcfutils (7) script was used to generate an intermediate consensus sequence in which any indels relative to the original reference sequence were appropriately called. The intermediate consensus was used as the reference for subsequent iteration of mapping and consensus calling. The total number of assembled viral reads was 87,971 (1.35% of the total reads). Despite the low proportion of viral sequence detected within the total data set, coverage of the entire genome was obtained (average read depth, 926.86×). The complete genome sequence was 11,922 nucleotides and most closely related to accesion no. LN879480.1, an RABV genome sequence from Republic of Azerbaijan. The present sequence represents the first full-genome sequence available for an RABV from the Republic of Tajikistan.

Described here is a simple methodology to successfully obtain a complete viral genome sequence from an FTA card without RNA extraction. The application of this methodology to other viruses could significantly aid the use of FTA cards to obtain viral nucleic acids without extraction for multiple uses, including molecular diagnostics and full-genome sequencing.

### Accession number(s).

The sequence data have been deposited in GenBank under accession number KY765901.
